# Reactivation of cereberal tuberculosis post-adalimumab therapy for rheumatoid arthritis: a case report

**DOI:** 10.1097/MS9.0000000000003450

**Published:** 2025-05-30

**Authors:** Adnan Alghazzawi, Zyad Al-Frejat, Marie Ahmad, Fadi Barkil, Lora Shammas, Enas Abood Darwish

**Affiliations:** Department of Neurology, Damascus university, Syria, Damascus, Syria

**Keywords:** adalimumab (anti-TNF), cerebral tuberculoma, rheumatoid arthritis

## Abstract

**Introduction and importance:**

Patients with rheumatoid arthritis who are treated with adalimumab have an increased risk of developing latent infections. The lethal risk of TB encephalitis as a potential manifestation after treatment with adalimumab should not be overlooked despite its rarity.

**Case presentation:**

We report a case of a 19-year-old Middle Eastern female who developed cerebral tuberculosis after receiving adalimumab therapy for rheumatoid arthritis. The patient was systemically well. Her medical history included pneumonia, PCOs (polycystic ovary syndrome), and *H. pylori gastritis*. Subsequently, she showed signs of anxiety after treatment with adalimumab. Magnetic resonance imaging (MRI) of the brain revealed a ring-enhancing lesion. An analysis of cerebrospinal fluid (CSF) failed to detect tuberculosis. The patient was treated and responded favorably to the tuberculosis standard four-drug anti-TB regimen (rifampicin, isoniazid, ethambutol, and pyrazinamide) and continued to show clinical improvement under ongoing treatment.

**Clinical disscusion:**

Rheumatoid arthritis patients who are treated with DMARDs are at risk of developing opportunistic infections. While most opportunistic infections are well understood and have clear symptoms, the rare occurrence of encephalitis should not be dismissed.

**Conclusion:**

Although rare, TB encephalitis should be considered in patients developing neurological symptoms after treatment with Adalimumab.

## Introduction

Rheumatoid arthritis (RA) is an inflammatory autoimmune disease characterized by chronic joint inflammation. Initially, RA affects small joints symmetrically and then progresses to larger joints^[^1^]^. RA manifests as fever, fatigue, weight loss, and stiffness of the affected joints for lasting more than 30 minutes. It is seen mainly in middle-aged individuals (35–60 years) and has a prevalence of 1% worldwide^[^2^]^. Extra-articular symptoms are not uncommon, affecting the eyes, heart, central nervous system, and other organs. Also, RA has the capability of affecting young populations < 16 years old known as juvenile rheumatoid arthritis which is similar to RA except for the absence of the rheumatoid factor^[^1^]^. The debilitating nature of this disease, alongside its chronic attributes, necessitates long-term treatments and a planned approach to try to stop its progression and enhance the quality of life for patients.

**HIGHLIGHTS**

- **TB Encephalitis After Adalimumab**: A 19-year-old with seronegative spondyloarthropathy developed cerebral TB six months after starting adalimumab treatment, presenting with severe neurological symptoms and brain MRI findings of tuberculomas.
- **Risk of Reactivating Latent TB**: Adalimumab, an anti-TNF drug, increases the risk of reactivating latent TB due to its immunosuppressive effects.
- **Difficult Diagnosis of CNS TB**: Despite negative CSF tests, the patient’s brain MRI and clinical response confirmed TB encephalitis, highlighting diagnostic challenges.
- **Successful Anti-TB Treatment**: The patient responded well to a standard 4-drug TB regimen, with significant clinical improvement and regression of tuberculomas on follow-up MRI.
- **Need for Monitoring Immunosuppressed Patients**: This case emphasizes the importance of monitoring for TB in patients on immunosuppressive therapies like adalimumab.

Currently, the main treatment approach is immunosuppression using corticosteroids or symptom relief using NSAIDs as first-line treatments. However, some cases of RA require a more aggressive approach using disease-modifying antirheumatic drugs (DMARDs). These drugs promote remission of the disease and are proven to reduce the risk of developing RA-associated lymphoma^[^3^]^.

DMARDs are a wide group of drugs with various mechanisms of action, such as but not limited to methotrexate (folic acid analog), hydroxychloroquine (works as an inhibitor of pro-inflammatory cytokines secreted by monocytes), and leflunomide (inhibits the synthesis of ribonucleotide uridine monophosphate pyrimidine)^[^3^]^. Another group of DMARDs that are used when the previously mentioned medications do not exhibit any improvement are tumor necrosis factor (TNF) inhibitors such as etanercept, infliximab, golimumab, certolizumab, and adalimumab^[^4^]^.

Adalimumab usage for RA is well documented, and it has shown great efficacy in treating the condition and preventing progression^[^5^]^. However, complications of using this drug are primarily seen due to its mechanism of action. Adalimumab inhibits TNF-α, a major proinflammatory cytokine that has a fundamental role in adaptive immune responses, which in turn may lead to increased susceptibility to severe and opportunistic infections or even reactivation of dormant infections^[^6^]^.

Tuberculous encephalopathy is a rare manifestation of the tuberculous infection, accounting for 3% of extrapulmonary infections^[^7^]^. The bacteria colonizes the brain after traveling via hematogenous spread from the primary site of infection in the lungs. The bacteria then induce an inflammatory process similar to the one it makes in the lungs that affects the brain parenchyma and the meninges as well, leading to granuloma formation, fibrosis, and even necrosis^[^8^]^.

This report presents a case of a female patient with RA who developed signs and symptoms of TB encephalitis six months after being started on adalimumab therapy, although reactivation of dormant infections and opportunistic pathogens is a well-established complication of immunosuppressants. Reporting these cases is fundamental for raising the attention level and helping with the early detection of these possibly fatal conditions.

## Case presentation

We are reporting a case of a 19-year-old Middle Eastern woman with a 9-month history of seronegative spondyloarthropathy (a subtype of serum negative RA) who was admitted to the hospital 4 months ago for pneumonia. The patient has a history of PCOs and H. pylori gastritis which she was treated for with the triple therapy of amoxicillin, clarithromycin, and omeprazole. Six months ago, the patient was started on 40 mg of adalimumab subcutaneously once every two weeks to treat seronegative spondyloarthropathy.

She was investigated for a 2-week history of severe, persistent, peri-orbital headache unresponsive to analgesics with forceful, projectile vomiting. At the beginning, she reported a bilateral pain in her eyes focused in the medial aspect of the right eye right eye that started 5 days ago, and bilateral weak and blurred vision. Shortly after, she experienced a fever (up to 39°C) on three occasions and grade 2 dyspnea on exertion with no cough. She also experienced loss of sensation in her hands and pain in her hips and shoulders.

Upon clinical examination, the patient had nuchal rigidity and a positive Kernig’s sign, and she exhibited arthritic pain in the large joints. She was severely agitated during the examination and was in a delirious state.

Her laboratory investigations revealed elevated WBC and ESR; other labs were within normal limits.

During her admission at the hospital, at night, she suffered from four episodes of hyperemesis.

The history of adalimumab administration alongside the high prevalence of TB in Syria. made TB the leading differential diagnosis, so a CSF sample was sent to the labs to investigate a TB infection.

**Figure 1.**
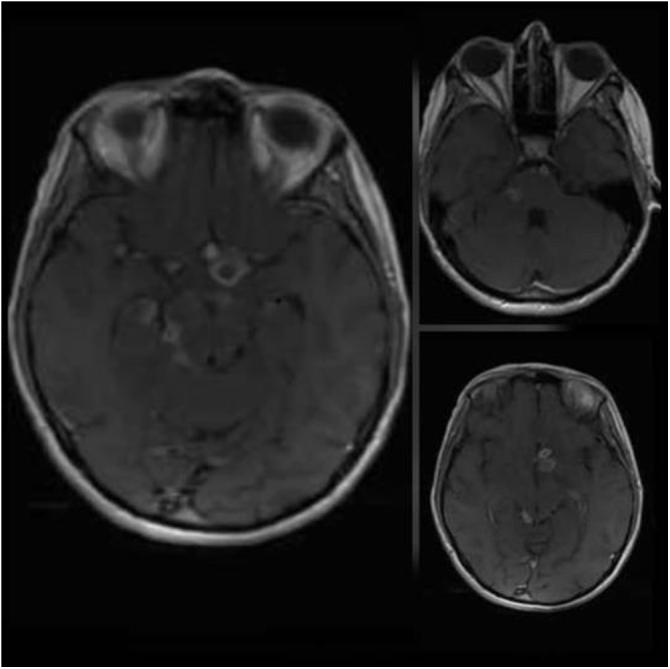
Axial T1-weighted MRI brain post-gadolinium demonstrating multiple ring-enhancing lesions.

**Figure 2.**
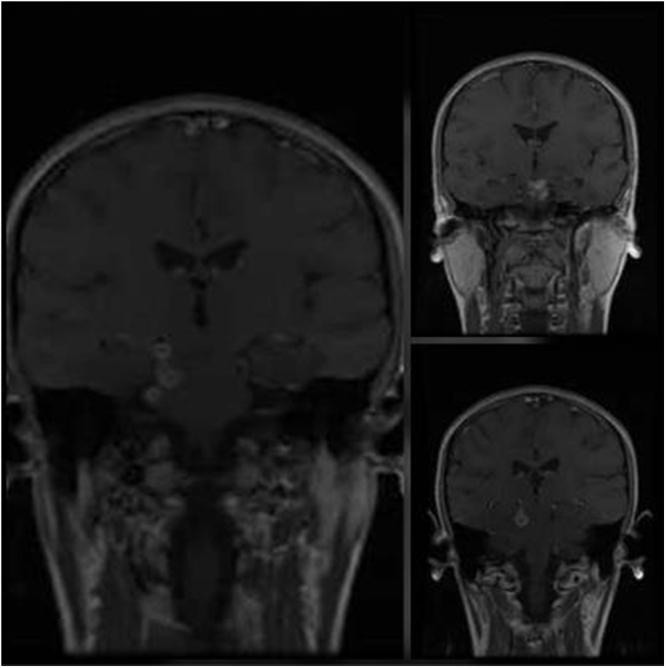
Coronal T1-weighted MRI brain post-gadolinium demonstrating multiple ring-enhancing lesions.

A polymerase chain reaction (PCR) and acid-fast bacilli (AFB) staining of CSF were negative for Mycobacterium tuberculosis. However, the high clinical suspicion for TB alongside the low sensitivity of these measures to detect TB in CSF (70% sensitivity) led to more investigations, so a magnetic resonance imaging (MRI) of the brain was ordered, and it showed many ring-enhancing lesions, suggesting multiple cerebral tuberculomas [Fig. (1), Fig. (2)].

Tuberculosis was the main differential diagnosis due to the lack of evidence of other possible diagnoses such as toxoplasmosis, cysticercosis, embolic abscesses, etc.

The patient was started on an antipsychotic (haloperidol) and four antituberculosis medications (rifampicin, isoniazid, ethambutol, and pyrazinamide). This led to the patient’s improvement in the morning, with no more agitation. The medications also included an anticoagulant (apixaban), an antipyretic (acetaminophen), an antibiotic (ceftriaxone), a corticosteroid, and an anticonvulsant (topiramate).

5 days after the patient’s admission to the hospital, she still had hectic fevers, so she was started on vancomycin as an additional treatment. As a result, on the sixth day, she had allergic urticaria due to the treatment with vancomycin, so vancomycin was stopped immediately, and hydrocortisone was prescribed for her.

**Figure 3.**
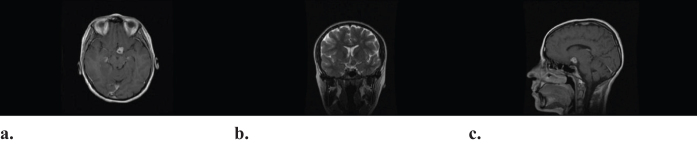
Latest MRI of the brain showing regression of tuberculomas in (**a**) axial T1-weighted MRI brain post-gadolinium, (**b**) coronal T2-weighted MRI brain post-gadolinium, and (**c**) sagittal T1-weighted MRI brain post-gadolinium.

She was kept on the standard therapy for TB and continued to show improvement clinically. Nine months later, a new MRI of the brain showed regression of the tuberculomas (Fig. 3).

## Discussion

Adalimumab is a human monoclonal TNF-alpha antibody given by subcutaneous injection, and it works by blocking the effects of TNF-alpha^[^9^]^. Despite its possible disease-modifying benefits through immunomodulation and immunosuppression, neutralizing TNF-α can also make individuals more vulnerable to infections^[^10^]^. Adalimumab increases the risk of serious infections such as reactivation of tuberculosis (TB) and deep fungal infections^[^9^]^. It hypothetically increases the possibility of infections with other pathogens such as bacteria (*Listeria monocytogenes* or *Salmonella* spp.) and viruses (hepatitis B virus, VZV, and human polyomavirus)^[^10^]^.

Only a minority of TB-infected patients are complicated by CNS TB (1% to 5%). Populations of countries that have a low prevalence of TB rarely show symptoms of extrapulmonary infection, the majority of which are reactivated diseases; among those, the predominant manifestation is TB meningitis^[^10^]^.

Central nervous system tuberculosis represents nearly 1% of tuberculosis cases worldwide. Most of them manifest as tuberculosis meningitides, and a minority as TB encephalitis, tuberculomas, and tuberculous brain abscess. People like children and HIV-infected patients are more likely to develop central nervous system tuberculosis than others^[^11^]^. Among patients with TB encephalitis, tuberculomas are considered the most common parenchymal appearance of brain TB. Multiple tuberculomas in immunosuppressed patients can be caused by diffusion from the bloodstream or by spreading through cortical veins and small penetrating arteries after a CSF infection^[^12^]^.

After hematogenous dissemination, these multiple or solitary tuberculomas can be located anywhere within the brain but are more expected to be located at the corticomedullary junction and periventricular region^[^13^]^.

Only a few cases of tuberculosis under anti-TNFα treatment have been described worldwide. Our case talks about tuberculosis involving the CNS occurring post-adalimumab treatment in the context of seronegative spondyloarthropathy, which may be considered one of the rare documented cases.

In patients with rheumatoid arthritis (RA) taking anti-TNF drugs, it is utterly unique to report a case of isolated NTB without other systemic manifestations. The identification of this case was based on the existence of skeptical symptoms^[^14^]^.

This can be hard since symptoms related to inflammation (e.g. fever) and matching signs in the lungs (pulmonary infiltrate or apical cavitation) may be absent, as well as its presentation is non-specific and may mimic other causes of chronic meningoencephalitis^[^15^]^.

Another way of diagnosing TB encephalitis is through biochemical analysis of the cerebrospinal fluid to investigate biomarkers. CSF lactate levels have been used as a diagnostic marker for central nervous system infections. Lactate is produced by bacterial anaerobic metabolism, and increased CSF levels have been reported in patients with bacterial meningitis and TBM. Clinical experience in Vietnam suggests that CSF lactate levels of 5–10 mmol/l support a diagnosis of TB, and that high initial levels are associated with death: in our case, a CSF sample was collected for the patient to test for gen-expert (PCR TB) to investigate for tubercle bacilli by smear examination or by bacterial culture. Standard staining techniques such as Ziehl-Neelsen, Kinyoun, or auramine-rhodamine applied to CSF samples have been estimated to detect approximately 100 AFB/ml of CSF and came back with negative results which are not definitive because the sensitivity of this procedure does not exceed 70% which means there is a chance 30% of a false negative result^[^16^]^.

Furthermore, the use of modern technology that the World Health Organization recommends is another promising, rapid, and cost-effective technique in resource-limited regions, which is used for molecular TB diagnosis in CSF specimens. Particularly in situations with limited sample volume, Xpert MTB/rifampin (Xpert MTB/RIF), a polymerase chain reaction technique to be used for molecular TB diagnosis in CSF specimens, is a quick and automated cartridge-based nucleic acid amplification next-generation technology that targets MTB’s rpob gene, bearing a sensitivity of 80.5% and a specificity of 97.8%^[^16^]^.

Reactivation of latent TB into active disease typically involves the weakening of the immune system such as HIV infection, old age, malnutrition, and medical conditions that compromise the immune system such as poorly controlled diabetes mellitus, renal failure, and therapy with immunosuppressive drugs, which are other factors that lead to immunodepression and reactivation of a dormant infection^[^17^]^.

Immunosuppressive drugs such as anti-TNF (adalimumab) are the main reason for this reactivation in our study due to the reduction in CD8 + responsible for the antimicrobial activity against *Mycobacterium tuberculosis*. After starting anti-TNF therapy, the risk of developing TB increases by 1.6 to 25 times. In TB-endemic countries, the risk of developing active TB in patients using anti-TNF is 10%. There is evidence that this occurs, on average, 12 weeks after starting treatment^[^18^]^.

The paramount diagnostic method in this study was settled retrospectively due to the impressive improvement in the patient’s overall status after providing her with quad max (which is a 4-drug fixed dose combination(FDA)) of anti-tuberculosis drugs including rifampicin, isoniazid, pyrazinamide, and ethambutol. The trial came back with a positive outcome.

The current guidelines recommend 4 orally administered drugs: isoniazid (INH) 5 mg/kg/d, rifampicin (RMP) 10 mg/kg/d, pyrazinamide (PZA) 25 mg/kg/d, and ethambutol (ETB) 15 mg/kg/d for the first 2 months^[^16^]^.

## Conclusion

Our report presents a case of a female patient with neurotuberculosis following treatment with adalimumab for rheumatoid arthritis (RA).

Latent tuberculosis typically remains asymptomatic; however, when immune suppression occurs, the infection can reactivate, presenting symptoms such as headache, nausea, and vomiting.

This underscores the importance of evaluating the serological status of any patient undergoing immunosuppressive treatment.

Furthermore, close monitoring and assessment of neurological symptoms and serological examinations are essential, regardless of primary infection status.

## Methods

The case has been reported in line with the Surgical Case Report (SCARE) 2023 criteria^[^19^]^.

## Data Availability

The datasets used and/or analyzed during the current study are available from the corresponding author on reasonable request.

## References

[1] McInnes IB Schett G. The pathogenesis of rheumatoid arthritis. N Engl J Med 2011;365:2205–19. 22150039 10.1056/NEJMra1004965

[2] Alamanos Y Voulgari PV Drosos AA. Incidence and prevalence of rheumatoid arthritis, based on the 1987 American College of Rheumatology Criteria: a systematic review. Semin Arthritis Rheum 2006;36:182–88. 17045630 10.1016/j.semarthrit.2006.08.006

[3] Bullock J Rizvi SAA Saleh AM. Rheumatoid arthritis: a brief overview of the treatment. Med Princ Pract 2018;27:501–07. 30173215 10.1159/000493390 PMC6422329

[4] Lis K Kuzawińska O Bałkowiec-Iskra E. State of the art paper tumor necrosis factor inhibitors – state of knowledge. Arch Med Sci 2014;6:1175–85. 10.5114/aoms.2014.47827 PMC4296073 25624856

[5] Perpétuo IP Caetano-Lopes J Rodrigues AM. Effect of tumor necrosis factor inhibitor therapy on osteoclasts precursors in rheumatoid arthritis. Biomed Res Int 2017;2017:1–10. 10.1155/2017/2690402 PMC5327780 28286757

[6] Tzanetakou V Stergianou D Giamarellos-Bourboulis EJ. Long-term safety of adalimumab for patients with moderate-to-severe hidradenitis suppurativa. Expert Opin Drug Saf 2020;19:381–93. 32098513 10.1080/14740338.2020.1734560

[7] Sotgiu G Falzon D Hollo V. Determinants of site of tuberculosis disease: an analysis of European surveillance data from 2003 to 2014. PLoS One 2017;12:e0186499. doi:10.1371/journal.pone.0186499. 29155819 PMC5695811

[8] Stahl JP. Tuberculous Encephalitis. Extrapulmonary Tuberculosis. Cham: Springer International Publishing; 2019:121–30.

[9] Scheinfeld N. Adalimumab: a review of side effects. Expert Opin Drug Saf 2005;4:637–41. 16011443 10.1517/14740338.4.4.637

[10] Martins F Rodrigues A Fonseca Oliveira J. Cerebral tuberculosis after therapy with adalimumab for hidradenitis suppurativa: a rare case. Cureus 2024;16:e52267. doi:10.7759/cureus.52267. 38222988 PMC10788140

[11] Tissot C Couraud S Meng L. Life-threatening disseminated tuberculosis as a complication of treatment by infliximab for Crohn’s disease: report of two cases, including cerebral tuberculomas and miliary tuberculosis. J Crohns Colitis 2012;6:946–49. 22749231 10.1016/j.crohns.2012.02.018

[12] Rodriguez-Takeuchi SY Renjifo ME Medina FJ. Extrapulmonary tuberculosis: pathophysiology and imaging findings. RadioGraphics 2019;39:2023–37. 31697616 10.1148/rg.2019190109

[13] Chaudhary V Bano S Garga UC. Central nervous system tuberculosis: an imaging perspective. Can Assoc Radiol J 2017;68:161–70. 28283299 10.1016/j.carj.2016.10.007

[14] Bortolozzo Graciolli Facanali C Roberto Facanali Junior M Vaz Safatle-Ribeiro A. Neurotuberculosis in a patient with ulcerative colitis using long-term adalimumab: a rare case. Am J Case Rep 2023;24:e938353. 36918754 10.12659/AJCR.938353 PMC10024933

[15] Torok ME. Tuberculous meningitis: advances in diagnosis and treatment. Br Med Bull 2015;113:117–31. 25693576 10.1093/bmb/ldv003

[16] Gupta M Tobin EH Munakomi S. CNS Tuberculosis. [Updated 2024 May 6]. In: StatPearls [Internet]. Treasure Island (FL): StatPearls Publishing; 2025. https://www.ncbi.nlm.nih.gov/books/NBK585138/36256788

[17] Ahmad s. Pathogenesis immunology, and diagnosis of latent *mycobacterium tuberculosis* infection. Clin Dev Immunol 2011;2011:1–17. 10.1155/2011/814943 PMC3017943 21234341

[18] Bruns H Meinken C Schauenberg P. Anti-TNF immunotherapy reduces CD8+ T cell-mediated antimicrobial activity against mycobacterium tuberculosis in humans. J Clin Invest 2009;119:1167–77. 19381021 10.1172/JCI38482 PMC2673881

[19] Sohrabi C Mathew G Maria N. The SCARE 2023 guideline: updating consensus Surgical CAse REport (SCARE) guidelines. Int J Surg 2023;109:1136–40. 37013953 10.1097/JS9.0000000000000373 PMC10389401

